# Lost in the loop - a qualitative study on patient experiences of care in standardized cancer patient pathways

**DOI:** 10.1186/s12913-023-10364-3

**Published:** 2023-12-07

**Authors:** Monica Solberg, Geir Vegard Berg, Hege Kristin Andreassen

**Affiliations:** 1 Innlandet Hospital Trust, Norway, Brumunddal and Norwegian University of Science and Technology, Gjøvik, Norway; 2 Inland Norway University of Applied Sciences, Elverum, Norway; 3 UiT The Artic University of Norway, Tromsø, Norway; 4 Norwegian University of Science and Technology, Gjøvik, Norway

**Keywords:** CPP, Cancer, Standardized pathways, Patient, Care, Qualitative, Experiences

## Abstract

**Background:**

The Norwegian health authorities introduced standardized cancer patient pathways (CPPs) in 2015, aiming to reduce practice variations across hospitals and regions, and improve the continuity, coordination and overall quality of the health care service provided to cancer patients. There has been few studies investigating this change, and that have looked into the organisational and economic benefits of standardized pathways, however the element of care and the patient perspective has been especially neglected. This study explored the care element in cancer patient pathways through an in-depth study of patient experiences.

**Methods:**

The patients were enrolled approximately three years after the introduction of standardized CPPs in Norway. Through a qualitative design with in-depth interviews, a total of 21 interviews were conducted with seven patients between 2018 and 2020. The first interview took place after the diagnosis was established and before treatment, the second interview during treatment, and the final interview approximately one year after the completion of active treatment. The empirical catchment area was eastern Norway. Data were analysed using a theoretical thematic analysis.

**Results:**

This study sheds light on the complex challenges patients’ faces, while navigating CPPs, including the need for better transition support, improved coordination and continuity in care, and a more holistic approach that encompasses emotional well-being and family support. Three overarching themes were identified: [1] Navigating CPPs: patient care and transition challenges, [2] Fragmented cancer care: challenges in coordination and continuity [3] Unmet needs and overlooked opportunities in CPPs.

**Conclusions:**

Patients experience that cancer patient pathways offer good medical treatment, but that the care element deserves more attention. Current CPPs are trapped in a logic of choice, preventing room for the element of care to receive the attention it requires for the patient to truly experience holistic person-centred care and continuous, well-coordinated services. Based in our study we argue there is a need to look into the missed opportunities for using the CPPs as points of departure for more holistic collaborative models for cancer care.

**Supplementary Information:**

The online version contains supplementary material available at 10.1186/s12913-023-10364-3.

## Background

Health authorities across Western countries have developed policy strategies and reforms based on visions of a more user-oriented health care service [1, 2]. Standardized pathways for specific patient groups, such as CPPs [2, 3] are part of the operational changes associated with this shift. The policy visions behind standardized pathways is to reduce practice variations across hospitals and regions, and to improve continuity, coordination and the overall quality of the health care service provided [4]. Over the last two decades CPPs have been implemented in the Scandinavian countries, beginning with Denmark in 2007 [5], and followed by Norway and Sweden in 2015 [3, 6]. The implementation of CPPs in the Scandinavian countries is an area of ongoing research and investigation. While initial findings suggest that CPPs can improve timely access to cancer diagnosis and treatment, reduce delays, and enhance patient outcomes, further systematic studies and long-term assessments’ are required to comprehensively evaluate the effectiveness and potential drawbacks of CPPs in the context of the Scandinavian health care systems [7–10]. According to a study done by Beau, Lynge [11] the estimated benefit-to‐harm ratio was 2.6 for invited women and 2.5 for screened women. Hence, 2–3 women would be prevented from dying from breast cancer for every woman overdiagnosed with invasive breast cancer or invasive and ductal carcinoma in situ (DCIS).

It is well-known that patients with cancer and their families do not only have physical care needs related to the disease and the treatment, but also a wide range of interpersonal, emotional and social care needs [12]. In 2008 the European Union (EU) [13] stated that:

…cancer treatment and care is multi-disciplinary, involving the cooperation of oncological surgery, medical oncology, radiotherapy, chemotherapy as well as psycho-social support and rehabilitation and, when cancer is not treatable, palliative care. Services providing care to the individual patient and support to the patient’s family must be effectively coordinated.

The concept of person – centred care strives to blend both objective and subjective viewpoints in order to attain a comprehensive understanding of illness and its associated treatment modalities [14]. This approach inherently recognizes the uniqueness of each individual and places as strong emphasis on considering their life experiences, values and personal preferences when delivering care. By practicing person – centred care, the potential exist for positive impacts on both emotional and psychological dimensions of an individuals’ wellbeing, ultimately fostering a profound sense of respect, dignity, and self-determination [14]. Users of health services often experience the care as fragmented, and deficient in emotional and psychological support, as well limited involvement in their treatment decisions [15, 16]. This becomes more comprehensible when we delve in the complexities of care [17, 18]. The integration of person-centred care principles is crucial in addressing this issues and aligning health care services with unique needs and preferences’ of each individual [19].

The aim of this study is to enhance our understanding of patients care experiences at three specific point in time along the CPPs. This study seeks to empirically explore how cancer patients themselves experience care in these particular settings.

### Theoretical approach

To enable us to identify and thoroughly reflect on patients’ experiences of care in CPPs, we found the care theories of Mol [20] and Tronto [21] useful. These two perspectives complement each other by offering a valuable synergy: Mol [20] provides a well-defined conceptual dichotomy, while Tronto [21] delves into the practical aspects of activities and moral perceptions.

Mol [20] outlines two contrasting main logics when she talks about different understandings of “good” care: the logic of care and the logic of choice. In a logic of care, the understanding is founded in the care practices, “what health care services do”, including both medical and social dimensions. For people working with care provision within a logic of care, their work is not about finding the shortest path from being sick to healthy, but rather about helping the person in need of care to find ways of dealing with life itself. If you are working within a logic of choice, on the other hand, this implies that you relate to and reflect more on the opportunities presented to the person you are caring for and on the choice that has to be made; “what are the choices available and what they choose” [20]. In a logic of choice, the health care service is restricted to assist the patient in making choices within a biomedical context. The social dimension is not included in the care work.

Tronto [21] provides us with what she describes as “a political ethics of care” where she describes how good care can be applied in practice. She identifies some integrated moral elements that are required to achieve “good” care. These elements are attentiveness (what are the care needs in any context?), responsibility (who should be responsible for meeting the needs for care?), competence (does the carer have the skills required to care?), and responsiveness (how far does care meet the needs of the cared for and the carer?) [21]. Tronto [21] defines care as:

…a species of activity that includes everything we do to maintain, contain, and repair our ‘world’ so that we can live in it as well as possible. That world includes our bodies, ourselves, and our environment [21].

In summary, Mol and Trontos theoretical perspectives offers a framework for this article as it aligns with the complex, individualized, and ethical nature of care in this context.

## Method

### Design

A qualitative approach was used, involving semi-structured interviews with patients conducted at three points in time throughout the cancer trajectory. This approach was useful to obtain experiences across time with the same informants [22]. Thematic analysis was selected as the method to identify, analyse and describe essential themes related to care in the patients’ stories. This approach allows us to form a systematic picture of what themes and events appear as significant for the patients experiences of care within a cancer trajectory [23]. Furthermore, this approach is especially useful for research that focuses on policy and practice [24]. The focus on the analysis is on the content of the telling – on what is told, not on how it is told [25]. The data set was a part of a larger corpus of data, produced in a research project on how a cancer diagnosis affects and is experienced by the patient and their family members through three phases of a cancer trajectory. For this study, all interviews from patient informants have been drawn out for a joint analysis. For an analysis of interviews from phase one only, see Solberg, Berg [15], and for an analysis of family members experiences, see Solberg, Berg [26].

### Setting

The study was conducted in one Norwegian health region and included one regional hospital. The regional hospital is organized into seven local hospital units covering an area of approximately 300 km.

### Recruitment and participants

The patients were recruited through an outpatient clinic, where they received their diagnosis. Initially, participants were given verbal and written information about the study and were invited to participate. The selection of patients was done using a strategic sampling approach. Those who accepted the invitation provided contact details for scheduling of meetings. Seven patients accepted the invitation (see Table 1), while six patients declined. Among the patients who did not wish to participate in the study, three of them cited a lack of energy and capacity as the reason, while the other three provided no specific feedback.

**Table 1:** Sociodemographic information about the participants

Patient	Age	Gender	Diagnosis	Treatment	How the cancer diagnosis was discovered
**Janne**	> 60	Female	Lung	Chemotherapy and radiation therapy	Was admitted to the hospital for another illness
**Laila**	51–60	Female	Breast	Surgery, chemotherapy and radiation therapy	Through mandatory mammography screening
**Susan**	41–50	Female	Breast	Surgery, chemotherapy and radiation therapy	Discovered a lump in the breast
**Sina**	51–60	Female	Breast	Surgery and chemotherapy	Discovered a lump in the breast
**Grethe**	> 60	Female	Breast	Surgery and radiation therapy	Was admitted to the hospital for another illness
**Lena**	51–60	Female	Breast	Surgery	Discovered a lump in the breast
**Iris**	51–60	Female	Breast	Surgery and radiation therapy	Through mandatory mammography screening

The patients who participated in the study had to meet the following inclusion criteria: they had to have a confirmed cancer diagnosis, speak and/or understand Norwegian, be capable of providing informed consent, and be over 18 years old.

### Data collection

Prior to the initial interview, two pilot interviews were carried out to assess the interview guides suitability for the study’s primary goals. These pilot interviews aimed to determine the appropriateness of the approach, as well as to evaluate potential themes and follow-up inquiries [22]. Both interviewees had experience in roles as caregivers to family members with cancer and as individuals who had personally received a cancer diagnosis.

Semi-structured narrative interviews were conducted in Norway by the first author between June 2018 and December 2020. This method was adopted since it is a recommended approach to build patients’ narratives on experience [27]. All of the interviews began with the open question “Can you tell me your story?” The open question was designed to elicit a narrative account and the interviewees were invited to speak as freely as possible (see Table 2). The interviews varied in terms of how the patients told their stories. Some told their stories without interruptions, while others needed more assistance. The patients were interviewed three times: before treatment, during treatment and about a year after active treatment (chemotherapy and radiation therapy).

**Table 2:** Information of opening question and themes from the interview guide

	First interview (before treatment)	Second interview (during treatment)	Third interview (about a year after treatment
Opening question	Can you tell me your story (from when you first suspected something was wrong up to now)?	Can you tell me your story (since the last time we spoke, up until now)?	Can you tell me your story (since the last time we spoke, up until now)?
Themes	Everyday life Family and network Quality of life and follow-up Information from the hospital	Everyday life Family and network Quality of life and follow-up Information from the hospital Experiences from meetings with the health care services	Everyday life Family and network Quality of life and follow-up Information from the hospital Experiences from meetings with the health care services

The interviews were conducted either in the home of the patients, in the hospital or via Skype, alone with the interviewer. The interviews lasted from 24 to 65 min per interview, and were digitally audio-recorded. All patients participated in all three interviews. The number after the name for each quote refers to interview number 1, 2 or 3.

### Ethical considerations

The research procedures in this study were approved by the Norwegian Centre for Research Data (ref. no. 51,466). The Regional Committee for Medical and Health Research Ethics in Norway (ref. no. 2016/1486) exempted the project from formal review since it was not anticipated to generate new knowledge about health and disease. Before the interviews were conducted, all participants received verbal information about the purpose of the study and that participation was voluntary. Study participants were also informed that they could withdraw from the study at any time without negative consequences and that they would have access to the collected data. To ensure confidentiality, a voice recorder was used to record the interviews. Each participant was assigned a number to ensure confidentiality. Anonymous transcripts, recordings, and a list of names with corresponding numbers were stored on a secure server in the healthcare facility, which only the project leader and interviewer had access to. In accordance with the protocol of the Norwegian Centre for Research Data, all collected data will be deleted at the beginning of 2024.

### Data analysis

In the analysis the interview material was examined trough a care–theoretical framework. This analytical approach enabled us to distinguish and highlight the care element in standardized CPPs. Mol’s [20] overarching distinction between a logic of care and a logic of choice and Tronto’s [21] concepts pointing to moral elements of care were combined to provide a framework suited to perform a detailed qualitative analysis of our patient interviews.

The thematic analysis process adhered to the six-stage procedure described by Braun and Clarke [23]: familiarization with the data, generating initial codes, generating themes, reviewing potential themes, defining and naming themes and producing the report. An inductive approach was used up to the third step, at which point a more deductive theory driven approach was embraced for the subsequent analysis. The rationale for this selection was the recognition that, subsequence to the identification of initial themes, it became evident that experiences related to the concept of care were a predominant pattern across the datasets.

To become familiar with the data, the first author read and re-read the transcribed material. Codes were generated while working with the interviews of individual patients. To capture the meaning in each individual’s narratives, the codes (units) were organized into conceptual themes. In the third step, a comparison of themes across the individual transcripts was conducted to identify similarities and differences. Then the themes, across the interviews, were defined and named as common themes. The thematic analysis revealed three main themes, further described in the result section.

The study was conducted in Norwegian during the interviews and data analysis, and the English translation was performed during the period of manuscript drafting. All words that could be removed without affecting the meaning of what was said, as well as some repeated words, were removed in the quotes. The names related to the quotes in this study is pseudonyms.

## Results

In the following section, the patients` experiences of care in CPPs is presented. The thematic analysis process yielded three main themes: [1] Navigating CPPs: patient care and transition challenges, [2] Fragmented cancer care: challenges in coordination and continuity [3] Unmet needs and overlooked opportunities in CPPs.

### Navigating CPPs: patient care and transition challenges

Our patients described how it was sometimes challenging to be on a CPP, because they experienced that there was a start and stop. Receiving a cancer diagnosis is a significant turning point in an individual’s life; for many, an experience of going from being a person to becoming a patient. The patients found that a dominant issue, in addition to having to cope with the cancer diagnosis itself and cancer-related issues, was managing life events that had happened before the cancer diagnosis, but that still required efforts and attention.

The patients we interviewed often started their stories by describing the setting and the context they were in when they first suspected that something was wrong, or when they received their cancer diagnosis. Some patients also mentioned events that were already having profound effects on their everyday lives prior to the diagnosis.

Susan was on sick leave with burnout syndrome and described her situation like this:

But what’s a bit depressing about the whole thing with my situation is that life’s thrown me a curveball … I’ve hit the wall, I was going to say, just before I found out about this, only three weeks before, so it was a bit much (crying) on top of it all (Susan – 1).

Another patient was on sick leave due to the loss of a close relationship, and described her situation like this:

I was in the middle of a grief process so, when I first got the diagnosis, it was like this: it just can’t be, it’s not possible, I can’t handle it now. So I don’t quite know if it has sunk in yet… there are some ups and downs, but sometimes I think that it (…) is very surreal. It can’t be right that I’m in, that it’s me who is in the middle of this here (Sina – 1).

Many of the patients said that the care they received was insufficient because their individual needs related to their particular context and situation were not met. One of the patients described the following:

There is no personal follow-up for me and my disease progression … However, I asked my GP if there were any physical therapists who could provide me with some advice, but that was not the case either … So, I spoke with some oncology nurses who said; “You can call and inquire about the possibilities”… It is my own merit that I actually received the offer (Sina – 3).

In our small sample, none of the patients had experienced that the healthcare personnel asked, talked about, or created opportunities for discussions on individual context and life in general prior to the diagnosis when talking with them. Lena said: *there was never a topic at the hospital. No one has brought it up (Lena – 2)*.

When it comes to experiences of follow-up after active treatment (chemotherapy and radiation), most of the patients told us that they had none, as no follow-ups had been offered. Lena’s story is an example. After surgery, Lena was told that there was no need for any aftercare in the form of chemotherapy and radiation treatment, she said: *I feel lucky that I did not need any further treatment (Lena-2)*, but that she unfortunately got burnout syndrome. Further, they told her that her “treatment is finished in terms of cancer”. Lena went on to describe her experiences of follow-up after treatment:

So, if I had still been on sick leave because I had to have chemotherapy or radiation, then in a way everyone would have understood that. I have great understanding from my doctor, but he doesn’t help me apart from giving me sick notes. But I feel I’m very alone in dealing with the situation I’m in, and it is quite tiring. I’m kind of tired of people asking how it’s going, and saying it’s not going so well (Lena-2).

Susan, however, had a different experience. She was the only one out of seven patients who had received information about relevant rehabilitation services after cancer treatment and had applied for this. *I think [rehabilitation services] has been the alpha and omega. I don’t know what my life would have been like without it (Susan-3)*.

As shown by the quotes and stories shared above, our interview material illustrates that the patient’s individual experiences of a cancer pathway must be understood in relation to the lives they lived before and after the treatment. However, healthcare personnel did not take the patient’s life situation prior to the diagnosis into account, and patients experienced a lack of follow-up or preparation for their life after active treatment.

### Fragmented cancer care: challenges in coordination and continuity

The patients described fragmented delivery of health care services, where cancer treatment is spread out across several hospitals and different departments in the health organization. The lack of continuity and collaboration was described on many levels: between members of the cancer team, between services in cancer teams and other departments where surgeries, tests and admissions are conducted and between providers in the hospital and the community health care services. Several of the patients had to deal with many clinicians and talked about the challenges this entailed. Janne said: *the way it is, there’s not much continuity there. I think I’ve talked to 13 doctors, or maybe it’s 14 (Janne-2)*.

The fact that there was little collaboration between with GPs and specialist health services, throughout the CPPs, led to frustration and uncertainty. The patients were informed that it was the GP who would follow them up after treatment, or if they had questions that were not related to the cancer treatment itself. Grethe explained why it was important that the GP was up to date:

Because it’s fine that the hospitals do their things, but it’s my GP who keeps me alive … because the hospitals only look at their part, while my GP has my whole history and everything that’s happened, and knows my story (Grethe-2).

The patients experienced that the lack of continuity and collaboration could lead them to be misinformed. In the first consultation, Sina received information about the benefits of removing the entire breast:

It was … yes, really caught me by surprise, because before the operation they sort of said that I would be spared that … seeing that they had to take the whole breast, then, that surgeon said, he said that … the advantage then is that you avoid both radiation and chemotherapy when we take it all (Sina-2).

In the consultation after the operation Sina received contradictory information that she had to have chemotherapy anyway. She continued by describing the consultation and how she reacted:

Reacted strongly to the way she said that… I think everything went completely black for me when she said I had to have chemotherapy… It was such an everyday thing for her [the doctor]. So it was not a good meeting. I was shocked to the core, actually (Sina-2).

Lena said that she experienced the care she received as managed by the individual function or department, and that there was little coordination. She gave an example:

First, I received a letter from the cancer department, and then another from the ones who were doing plastic surgery. Then came to two different appointments and two co-payments. Nothing fitted together … but the appointments were coordinated, so that fitted together (Lena-2).

The above quotes show that the patients did not experience continuity and collaboration within and between the health care services in their CPPs. The patients asked questions about the caregiving competence of the health care services when cancer care is provided in several units and departments. The fragmentation of services and the high number of people involved in care-provision contributed to fragmentation also of responsibility.

### Unmet needs and overlooked opportunities in CPPs

In the interviews, patients shared a range of experiences with healthcare services, both positive and negative. While many praised the competence of healthcare personnel in addressing physiological and medical aspects, they also highlighted issues they saw as “lost in the loop”. Janne expressed how she experienced the medical treatment: *Because they [health personnel] have been amazing over there at the cancer unit, they have been very sympathetic and friendly all the time (Janne-3).*

Patients generally trusted clinicians’ expertise in medical treatment and followed their recommendations, emphasizing the importance of specialized knowledge in cancer care decisions. *I have to trust that they [clinician] make the right decisions. After all, they have a long education*, said Sina when she talked about making choices in medical treatment. Janne had a similar answer:

… I hadn’t had any basis for saying whether I wanted chemotherapy or not. There are others who have expertise in that area. So I just accept that, because they have a lot of experience in how chemo works. So I wouldn’t have been in a position to make any comments about that. So I don’t think that makes any difference to me, if I had been asked about it (Janne-3).

However, negative experiences emerged as well. Susan described a disheartening encounter with a clinician who responded sarcastically to her husband’s inquiry about a broader examination. Susan’s reaction to the answer from the clinician was:

Yes, for my part, I thought, you [the physician] care so little. You have actually given a message that kicks one’s legs out from under one [the patient]; also, that question was asked by a completely inexperienced [person]. Then one feels, ‘my God, I’m not in good hands, I’m just a piece in the game, a piece in his job’ (Susan-1).

On the psychological and emotional front, patients expressed a sense of isolation as they navigated their cancer journey, with little guidance on where to seek emotional support. Sina said: *I have felt very alone … when you are discharged from the hospital after the operation, you are sort of on your own (Sina-2).*

The patients perceived a gap in providing care for their family members, who they felt were also left adrift in the “cancer loop”. *They [the family members] have received very little follow-up or no follow-up. It’s only if I have brought them along myself*, said Laila (Laila-3). Several patients stressed the significance of including family members in the cancer trajectory, acknowledging the vital role these loved ones play in their cancer experience. Susan said:

I understand that I am the patient and I have first priority, but still there is a quite a big difference, there is a quite a difference between those who kind of only see you and those who see us as a whole. Because that is very important (Susan-2).

Over time, patients recognized the importance of family involvement, even if some initially hesitated to burden their loved ones. Sina explained that at the beginning she did not want the attention of others because she did not want to be a burden. She said:

It’s a big strain, because then I have to feel it again and again and again, so I have kind of been dreading it and it’s tiring to get the feedback, which of course is only well-intentioned, but it does something to you all the time, because then reality hits you back all the time, so, is it so serious, … much easier to just be in my own little bubble (Sina-1).

Another patient expressed involvement of the family like this: *In hindsight, I see that as only positive … that support from the family means a whole lot (Iris-2).*

Patients present examples illustrating how the family members can be provided with support. One such example involves the recommendation that patients include a family member during their consultation, thereby ensuring the concurrent transmission of relevant information, and giving families the opportunity to ask questions. Laila said:

…at times I’m sure they also need information, but I don’t think they have received that. There are probably many questions for them too, and worries. There are things I don’t manage to communicate … (Laila-2).

In general, the above quotes highlighted the need for a more comprehensive approach to patient care that encompasses emotional well-being and family support, in addition to the traditional biomedical focus of cancer care. Despite these challenges, patients still acknowledged the quality of medical treatment they received and the trust they had in their clinicians.

## Discussion

The findings presented in this study provide insight into how patients, after undergoing standardized CPPs, experienced care in the cancer trajectory. They openly and articulately talked about their experiences, and they described stories relating to how they experienced care from the health care services in the different phases of the cancer trajectory. Throughout the CPPs, the patients experienced a wide range of challenges that were linked to physical, social and psychological consequences of life events that occurred before the diagnosis, but also from the fragmented provision of care. Overall, our analysis reveal that patients experience fragmented rather than holistic care, in spite of the political vision of standardized pathways as tools for continuity and coordination in care provision.

### Disrupted life events before cancer diagnosis

Bury (1982) introduced the concept of illness as “biographical disruption” [28]. When facing serious chronic illness, like cancer, “disrupted life courses” might develop when individual expectations for the future are not met [28, 29]. According to Annemarie Mol, care is insufficient when health care services do not take patients’ daily lives into consideration, and when physical parameters are isolated from their context [20]. This approach is also in line with Tronto’s framework, where the moral element of responsiveness is decisive of whether a caring process comes full circle [21].

The limited time frame of CPP does not seem to be compatible with a logic of care where both the social and medical dimensions of life are being taken care of. In our data, we found that some of the patients’ stories begin with them having disruptive life events even before the cancer diagnosis. Nevertheless, none of the patients in our sample had experienced that their life situation/event prior to the diagnosis was discussed or addressed as an issue. One of the reasons for this may be that the standardized pathway does not leave room for this concern. These findings are consistent with those from studies by Kvæl, Hellesø [30] and Salamonsen, Kiil [29], who conclude that there is a gap between the services the health care systems offer and the patient’s individual needs. On a system level this means there are challenges with providing person -centred care; who is responsible for meeting the patients’ various needs. Salamonsen, Kiil [29] further underline that addressing life events that form the patient pathway should be a priority. The logic of care, according to Mol, is an attempt to contribute to improving health care on its own terms and language. The main emphasis is not on the right to decide for oneself or autonomy, but on daily life practices. From what Mol describes as a “logic of care”, care is insufficient when patients are neglected, and when there is not enough time to listen to their accounts [20]. Further, our findings also illustrate that patients did not experience attentiveness and responsibility from healthcare professionals after starting on a CPP – two of the elements required for good care according to Tronto [21].

According to the guidelines for CPPs, there are certain topics that must be addressed in the first conversation after a person is diagnosed with cancer [31]. The guideline states: The conversation clarifies the patient’s expectation of the trajectory. The patient’s life situation/event, possible anxiety, resources, needs and wishes are uncovered [31]. The guideline in itself thus paves the way for what Tronto talks of as an element of “attentiveness”; mandatory for the needs of the cared for to be recognized and met in a caring relationship [21]. To achieve this, the carer has to take into account the uniqueness of each person by confirming that everyone has different life stories, experiences and perceptions [21, 32]. However, previous studies suggest that this discrepancy between policy guidelines and actual practice that we also observe in our study is neither surprising nor unusual [33, 34].

### Fragmented treatment offer

The Western health care system is highly specialized and fragmented, with patients often having to integrate consideration for all their conditions themselves. For patients who are already struggling, such health care systems can become an additional burden [35, 36], which we also observe in our study. Our findings show that the patients experienced that lack of continuity and collaboration within and between the health care services led to contradictory messages regarding information about medical treatment. Furthermore, the patients had to deal with several health care personnel. This led to patients becoming unsure whether they were getting the best treatment available.

The standardized pathways stimulate routines and reduce waiting-times for diagnosis and treatment, but threaten an approach guided by individual needs and preferences of both patients and professionals [4]. Furthermore, it has been argued that protocol-based care provokes, and reduces levels of active patient engagement during consultation [37]. According to Mol, “good” care has little to do with “patient choice”, but good care is something that grows out of collaborative and continuing attempts to adapt knowledge and technologies to complex lives and diseased bodies [20]. Our findings can well be interpreted to support this critique. The patients we talked to underlined how the health service offering standardized CPPs to cancer patients had low responsiveness to the individual needs of the cared for.

In the literature, it is discussed whether it is possible to combine individualization with standardization in cancer care [29, 30, 38]. In a meta-ethnography study, Kvæl, Hellesø [30] identify an urgent need for a coordinated conceptualization of the experienced tension in balancing standardization and individualization in CPP to ensure better health care services for patients and health care personnel.

### Several gaps in cancer care

Western health authorities [1, 2, 39] want patients to have a more active role in their own treatment and in the preparation of the health services. During the 20th century, health care services have shifted their approach from a traditional, paternalistic, disease-focused approach towards one that fully integrates the patient’s experiences and needs. The patients in this study experienced a healthcare service focused mainly on physiological issues (medical treatment). Our results show they experience good medical treatment, but find that other aspects of care such as (a) psychological and emotional care (b) care for the family and (c) individual health care needs, are not covered by health care personnel involved in the standardized CPP. Furthermore, our research revealed that patients were not offered follow-up care after the conclusion of active treatment. This absence of follow-up care is not merely an oversight but is a consequence of the organizational structure of the CPP.

According to a mapping review [40] in the oncology field, several gaps in the handling of psychological support for patients have been described. The authors have developed a new method that allows patients to report their perceptions of the care that was provided at multiple time-points along the care pathway [40]. To achieve improvements in experiences of cancer care components and phases, it is important to ensure the care provided is person-centred and aligns with patients’ preferences for component of care [40, 41]. Findings in a narrative review by Turchi, Dalla Riva [12] showed that the health care system should integrate care of the body with care for anything that is generated by an oncological diagnosis in psychological and interactive terms. This is in line with what Annemarie Mol calls a logic of care, which concentrates on all kinds of activities that the patient is engaged in [20].

Findings in this study illuminated that some patients experienced that health care personnel did not take them seriously, and this led to uncertainty about the knowledge and skills of health personnel. These findings are consistent with a study conducted by Avestan, Pakpour [42], who found that the dignity of cancer patients was not well respected, and the quality of the communication remained in a moderate level. According to Tronto, the patient and the family member will not experience caring if the health personnel fail to recognize their physical, psychological, cultural, and spiritual needs [21].

When it comes to physiological care, findings in this study show that patients want the clinician to make decisions for them, and they trust their choice of treatment. This is in line with other studies [43]. Nevertheless, this study does not elucidate whether this preference arises from a comprehensive understanding that empowers them to make informed choices, or if it is influenced by constraints inherent to the standardized patient pathways. An important condition is that it cannot be expected that a sick person will always be able to make conscious choices, but this does not mean that they can be treated as an object [20, 44]. Mol [20] states that when you are a patient you are not always able or in a position to make your own choices, and may well want health personnel to make decisions for you. She further argues that if a healthcare system is structured around a logic of choice, it could transform individuals into patient-consumers, burdening them not only with an option but also with a responsibility to decide. Consequently, this shifts the burden of any mishaps onto the patients shoulders [20].

The ”what matters to you” campaign in health care [45] argues that asking questions to find out what really matters to the patients is a great opportunity to improve quality in service delivery, and that the question “What is important to you?“ should guide the meeting between patient and health personnel and in the preparation of the health and care services, and this must contribute to creating the patient’s health service [46]. This initiative represents a global movement centred on person-centred care and is marked annually on either the 4th or 6th of June in more than 49 countries [45, 47]. The question creates new openings for empathy, seeing the person behind the patient, and realizing in a more thoughtful way what matters to patients at both a system and an individual level [45]. Nevertheless, Anderson, Spanjol [48] point out that this shift entails a risk that the practical involvement initiatives end up fostering “responsibilized” rather than empowered patients.

According to the results of this study and other recent studies [30, 49], support and care for the family members are lacking in current standardized patient pathways.

### Study limitations and strengths

This study has both strengths and weaknesses. To assess the quality of qualitative research, Lincoln and Guba [50] have proposed the concept of trustworthiness with the following criteria: credibility, dependability, and transferability. In this study, we conducted three interviews with the same informants at different stages of the patient trajectory—before, during, and after active treatment. This approach can be seen as both a strength and a challenge. Through repeated interviews, the researcher have the opportunity to explore the informants’ experiences in more depth and capture nuances that may not have been evident in a single interview. In this study, we view this method as a strength as it has provided us with a more comprehensive understanding of patients’ experiences of care throughout the patient trajectory and has helped identify areas where care practices can be improved. A challenge associated with this approach is the potential dropout of informants when conducting multiple interviews with the same individuals [51]. However, in this study, we did not experience any dropout among the informants.

The dependability of our findings was strengthened by the fact that all interviews were conducted by the same interviewer (MS). The interviewer was a female doctoral student and a registered psychiatric nurse with extensive experience and knowledge in conducting conversations with individuals in vulnerable situations. To further enhance dependability, we used a semi-structured interview guide with the same introductory questions in all three interviews, ensuring that all informants were asked the same questions. We included quotes in the presentation of the results, which strengthens the trustworthiness of our findings. The analysis was conducted collaboratively with all co-authors, contributing to increased transparency and reduced the risk of personal bias.

This study also has some limitations. The sample size was limited, and only two types of cancer are represented. Despite the small sample size, this study incorporated the experiences of patient of differing ages with different experiences of having a cancer diagnosis, and so constitute a relatively heterogeneous sample. This study thus provides an important in-depth insight into the experiences of the seven patients who participated.

### Implications for policy, practice and research

The findings of this study carry important implications for shaping healthcare policy. These findings emphasize that policymakers must actively consider how to better integrate the care element into standardized CPPs. This may require them to revise guidelines and protocols to ensure that the care aspect assumes a more central role. Policy should prioritize delivering comprehensive care that encompasses both the medical and the social/psychological aspects of patient treatment.

Furthermore, these findings stress the need to enhance the coordination and continuity of care, both within healthcare services and among different service departments and GPs. Achieving this demands a shift in attitudes and practices to offer patients more comprehensive care that fully embraces their social and emotional needs. Practice should go beyond simply selecting treatments and focus on creating a sustainable path for patients.

Additionally, research in cancer care should proactively explore and document how to seamlessly integrate the care element into standardized CPPs. This may involve conducting studies on enhancing patient experiences through care interventions, evaluating diverse approaches to care coordination and continuity, and delving into patients’ unique perspectives and needs. What is urgently required is a broader comprehension of the criteria for defining quality cancer care.

## Conclusion

The analysis of this study revealed that there is still a need for better continuity and coordination within and between departments and GPs. In sum, patients experience the care element as missing in their meetings with public health care services. Our conclusion is that current CPPs are trapped in a logic of choice, preventing room for the element of care to receive the attention it requires for the patient to truly experience person-centred care and improved continuity and coordination of the service.

### Electronic supplementary material

Below is the link to the electronic supplementary material.

**Supplementary Material 1: **ISSM_COREQ_Checklist

## Data Availability

The dataset generated from the study will be publicly available upon reasonable request to the corresponding author [MS] once anonymization of the data has been completed.

## Abbreviations

CPP: Cancer patient pathway
CPPs: Cancer patient pathways
GP: General practitioner
GPs: General practitioners
